# The Punishment for the Crime of Producing Abortions

**Published:** 1893-09

**Authors:** 


### THE PUNISHMENT FOR THE CRIME OF PRODUCING ABORTION.

This crime has been particularly rampant in this city in the past year. A statement of the punishment accorded to it in various countries of Europe may therefore prove interesting. In England and Ireland the punishment is penal servitude for life, or a less term. Should the mother die, the crime becomes murder, which may be punished by death. In Scotland (says *The Lancet*) the punishment is arbitrary; in France, Spain, the German Empire, Austria, Hungary, Italy, Russia, Norway, Sweden, and Denmark—in short, throughout the whole of Europe—the crime is punished with imprisonment for from six months to twenty years, or for life. In Sweden the penalty is death if the mother dies; and in Russia the mother, if a consenting party, may be exiled to Siberia; in the Dominion of Canada the penalty is imprisonment for life; in Nova Scotia, Quebec, Ontario, British Columbia, and in Prince Edward Island it varies from imprisonment for two years to for life; in New Brunswick the penalty is death; in Australia and New Zealand the punishment is very severe, ranging from two years' imprisonment to penal servitude for life; in the United States it is punished with fines ranging from $100 to $5,000, with imprisonment for long periods, and with death.

There is, as will be seen, a very general unanimity of view regarding the high degree of criminality of the practice. Despite all this, the crime is increasing both in our country and in Europe. There is no doubt that this is due to the fact that many more women than formerly, know of the possibility of abortion and refuse to accept the consequence of indiscretion or the responsibilities of maternity. There is but one way to look at it, however—abortion involves the destruction of one life and danger to another. Hence it is a crime never to be justified even under extreme circumstances.

It is true that many obstetricians still regard the induction of premature labor, even before the infant is viable, as a necessary measure in certain very rare cases. But the indications for it are becoming more and more restricted as medical science advances, and it is not improbable that the time is near at hand when the operation will no longer be thought of as a means of saving life. This legitimate obstetrical procedure, however, has nothing in common with criminal abortion, and medical men owe it to the community as well as to their profession to assist in limiting the work of those whose trade is infanticide. The daily papers could, doubtless, do more than any other agency toward abolishing the crime if they would consent to give up the small fees they get for advertising this class.—*New York Medical Record*, August 19, 1893.

